# BREAST-Q-Based Survey of the Satisfaction and Health Status of Patients with Breast Reconstruction

**DOI:** 10.1007/s00266-023-03642-2

**Published:** 2023-09-11

**Authors:** Lina Jiang, Xiaohui Ji, Wei Liu, Chuanchuan Qi, Xiaomei Zhai

**Affiliations:** 1 Deparment of Plastic Surgery, The first affiliated hospital of zhengzhou university, No. 1 East Construction Road, Jinshui District, Zhengzhou, 450052 China; 2 Department of Pathology, The people’s Hospital of Zhengzhou, Zhengzhou, China; 3 Department of Breast Surgery, The people’s Hospital of Zhengzhou, Zhengzhou, China

**Keywords:** Breast cancer, Quality of life, Mastectomy, Reconstructive surgical procedures, Patient satisfaction, Health status

## Abstract

**Aims:**

To explore the patients’ satisfaction and health-related quality of life (HRQOL) of patients who received reconstruction after breast cancer surgery using the BREAST-Q questionnaire and further investigate the influencing risk factors.

**Methods:**

This cross-sectional study enrolled patients who underwent first-ever breast reconstruction after unilateral or bilateral mastectomy at the Breast Surgery Department of First Affiliated Hospital of Zhengzhou University or People’s Hospital of Zhengzhou between January 2016 and December 2021. Multivariable linear regression analysis was used to analyze the risk factors.

**Results:**

A total of 202 participants were included. Age of >45 years (vs.≤35 years, *β *= − 3.74, *P *< 0.001) was an independent risk factor influencing the satisfaction degree score. Age between 36 and 45 years (vs. ≤35 years, *β *= − 0.26, *P *< 0.001), age of >45 years (vs. ≤35 years, *β *= − 0.45, *P *< 0.001), nipple-preserving mastectomy (NSM)/ skin-preserving mastectomy (SSM) + sentinel lymph node dissection + prosthesis implantation + contralateral breast augmentation (vs. NSM/SSM + sentinel lymph node dissection + prosthesis implantation, *β *= − 0.16, *P*=0.012), and the use of small intestinal submucosa (SIS) matrix (*β *= 0.13, *P *= 0.044) were independent risk factors influencing the HRQOL scores.

**Conclusion:**

Age, the surgical procedure, and the use of matrix were associated with the satisfaction degree and HRQOL after breast reconstruction in patients receiving mastectomy.

**Level of Evidence II:**

This journal requires that authors assign a level of evidence to each article. For a full description of these Evidence-Based Medicine ratings, please refer to the Table of Contents or the online Instructions to Authors www.springer.com/00266.

**Supplementary Information:**

The online version contains supplementary material available at 10.1007/s00266-023-03642-2.

## Introduction

Breast cancer is the most common malignancy diagnosed in women worldwide, with estimated new cases of 2,261,419 in 2020 [1, 2]. Early or resectable breast cancer, which is considered potentially curable, includes stage I-IIB and some stage IIIA cancers, specifically T3, N1 tumors [3]. The prognosis of breast cancer is generally satisfying, with a 5-year survival rate of 99 and 85% for patients with localized disease and regional spread, respectively [2]. Surgery is the mainstay treatment of breast cancer, but it will inevitably lead to cosmetic breast defects or missing breasts [4]. The breast is essential to body image for women, and many of them would have to live their entire life with an impaired body image [5, 6]. Hence, breast reconstruction surgery is an option to correct the shape of the breast after lumpectomy or reconstruct a breast after mastectomy [4, 7].

Depression is a major threat to the quality of life (QOL) of women with breast cancer since it deteriorates the patients’ somatic symptoms, decreases general functioning, and can even compromise adherence to treatments [8]. The proportion of women with breast cancer and depression was estimated at 11–20% [9]. Patients who did not undergo plastic surgery after mastectomy can have higher levels of depression and loneliness as well as poorer physical, social, and emotional functioning [10–12]. Breast reconstruction has been shown to improve mental health, stress, loneliness, and anxiety, but at the cost of higher physical discomfort and perceived physical distress [13]. A meta-analysis also concluded the uncertainty of the actual benefits brought by breast reconstruction to women [14].

Therefore, additional studies are necessary to find out the actual impact of breast reconstruction on the mental and physical health outcomes of women. The BREAST-Q is a patient-reported outcome tool that can be used to quantify the impact and effectiveness of breast surgery, including a questionnaire specific to reconstruction [15, 16]. A meta-analysis also supported the value of the BREAST-Q in measuring patients’ satisfaction and health-related QOL (HRQOL) after oncoplastic surgeries [17]. Furthermore, it is crucial to investigate the acceptance and postoperative satisfaction of patients, particularly regarding prosthesis reconstruction, in regions with high incidence of breast cancer and with conservative ideology. By understanding the specific factors that influence satisfaction and HRQOL, clinicians can work toward improving the popularity and effectiveness of prosthesis reconstruction in patients who have received breast mastectomy.

Hence, this study aimed to explore the satisfaction and health status of patients who received reconstruction after breast cancer surgery using the BREAST-Q questionnaire and further investigate the factors influencing the patients’ satisfaction and HRQOL. The results could help improve the management of women with breast cancer.

## Subjects and methods

### Study design and participants

This cross-sectional study enrolled patients who underwent first-ever breast reconstruction after unilateral or bilateral mastectomy between January 2016 and December 2021 at the Breast Surgery Department of First Affiliated Hospital of Zhengzhou University or People’s Hospital of Zhengzhou. The study was approved by the Ethics Committees of the First Affiliated Hospital of Zhengzhou University or People’s Hospital of Zhengzhou. Written informed consent was obtained from all participants.

The inclusion criteria were: (1) age of ≥ 18 years; (2) diagnosed with primary breast cancer according to the Guidelines of the Chinese Society of Clinical Oncology on Breast Cancer (2021 version) [18] and received surgery; (3) underwent reconstruction after breast cancer surgeries, using implantation materials (TE/Imp), latissimus dorsi flap, or transverse rectus abdominis myocutaneous flap (TRAM); (4) volunteered to participate in this study and complete the questionnaire survey. The exclusion criteria were: (1) prosthesis-related infections or ischemic necrosis of the flap; (2) tumor relapse or distal metastasis; or (3) incomplete baseline clinical data or follow-up.

### Questionnaire

The questionnaire was self-designed by the investigators after reviewing relevant studies and the medical records of patients. The questionnaire collected demographic characteristics and disease-related information. The demographic characteristics included age of disease onset, marital status, family income, and body mass index (BMI). The disease-related information included other underlying diseases (e.g., hypertension and thyroid diseases), radiotherapy, chemotherapy, targeted therapy, endocrine therapy, axillary lymph node dissection, tumor stage, molecular classification, surgical mode, breast volume, and timing of reconstruction. The breast cancer-related clinical information was collected by reviewing the medical records of the patients to guarantee the accuracy of the data.

## BREAST-Q scale

The BREAST-Q is a validated patient-reported scale that assesses the HRQOL and satisfaction degree of patients before and after breast reconstruction and plastic surgery [15]. The satisfaction degree part includes the satisfaction degrees regarding the breast, information, and medical team. The HRQOL part includes social psychological health, body health of the chest and upper limbs, and sexual health. The scoring system of BREAST-Q ranges from 0 to 100 points according to the performances of patients in different dimensions, and higher scores indicate higher HRQOL or satisfaction degree. The Chinese version BREAST-Q scale was used in this study, of which the overall internal consistency coefficient of the five modules was 0.912–0.980, and the internal consistency coefficient of a single dimension in each module was 0.741–0.978, indicating its high validity and reliability [19]. The questionnaires with less than 2/3 of the questions completed, or all the questions that were replied to by the same choice, were considered invalid and excluded.

### Statistical analysis

SPSS 26.0 (IBM, Armonk, NY, USA) was used for statistical analysis. The continuous data were all with normal distribution according to Kolmogorov–Smirnov test; they were described as means ± standard deviations and compared using analysis of variances (ANOVA). Categorical data were described as numbers and percentages. Paired-sample t test was used in the comparison between before and after surgery. Data among the three age subgroups were compared using one-way ANOVA test. For the multivariable linear regression analysis, satisfaction degree and HRQOL were used as the dependent variables, and the baseline characteristics with statistical significance were used as the independent variables. Two-sided P-values <0.05 were considered statistically significant.

## Results

### Characteristics of the participants

A total of 213 participants were enrolled. One patient was excluded for prosthesis-related infection, one for flap ischemia, and nine for invalid questionnaires. The baseline characteristics, satisfaction degree, and HRQOL are shown in Table 1. The satisfaction degree of the participants was significantly lower with increasing age (*P *< 0.001), increasing BMI (*P *= 0.001), higher TNM stages (*P *< 0.001), with radiotherapy (*P *< 0.001), with chemotherapy (*P *< 0.001), without neoadjuvant chemotherapy (*P *= 0.001), and different surgical approach (*P *= 0.027) (Table 1). The HRQOL was significantly lower with increasing age groups (*P *< 0.001), higher TNM stages (*P *= 0.012), without radiotherapy (*P *< 0.001), with neoadjuvant chemotherapy (*P *< 0.001), with different surgical approaches (*P *= 0.034), longer scars (*P *= 0.019), without flap harvesting (*P *= 0.014), and the use of small intestinal submucosa (SIS) matrix (TiLOOP Product, Pfm medical titanium gmbh, Germany) (*P *= 0.004) (Table 1). The comparison of satisfaction degree score between before and after surgery and among the age subgroups was presented in supplementary Table 1 and 2. “Satisfaction with Breasts,” “Psychosocial Well-being,” “Satisfaction with papilla,” and “Sexual Well-being” showed significant decrease after surgery compared to that before surgery as well as in all age subgroups (all *P *< 0.001). “Physical Well-being: Chest” significantly increased after surgery than that before surgery in all age subgroups (all *P *< 0.001). “Satisfaction with Breasts,” “Psychosocial Well-being,” and “Sexual Well-being” before surgery were significantly different among the three age subgroups (all *P *< 0.001). After surgery, “Satisfaction with Breasts” (*P *< 0.001), “Psychosocial Well-being” (*P *< 0.001), “Physical Well-being: Chest” (*P *< 0.001), “Sexual Well-being” (*P *< 0.001), “Satisfaction with the surgeons” (*P *< 0.001), “Satisfaction with the medical team” (*P *< 0.001), “Satisfaction with papilla” (*P *= 0.040), “Satisfaction with the information” (*P *= 0.006), and “Satisfaction with the other medical staff” (*P *= 0.001) were significantly different among the three subgroups, except for “Satisfaction with the prosthesis” (*P *= 0.073).

**Table 1:** Characteristics of the patients

Characteristic	*n* (%)	Satisfactory degree score	*P*	HRQOL	*P*
Total score		206.5±18.6		73.6±10.6	
Age			<0.001		<0.001
≤ 35 years	65 (32.2)	214.3±13.9		78.2±11.4	
36–45 years	84 (41.6)	207.1±18.9		73.6±9.4	
> 45 years	53 (26.2)	196.1±18.3		67.9±8.7	
Educational level			0.190		0.098
Primary school or junior middle school	38 (18.8)	203.0±18.0		71.0±11.5	
Senior middle school or higher	164 (81.2)	207.3±18.6		74.2±10.3	
Marital status			0.117		0.203
Married	19 3 (95.5)	206.1±18.7		73.4±10.7	
Unmarried	9 (4.5)	216.0±12.5		78.00±7.3	
Residence			0.436		0.249
Towns or cities	116 (57.4)	207.4±20.3		74.34±10.9	
Rural area	86 (42.6)	205.3±15.9		72.59±10.1	
Body mass index			0.001		0.498
< 18.5 kg/m^2^	5 (2.5)	215.8±6.7		79.00±14.3	
18.5–24 kg/m^2^	143 (70.8)	209.3±16.3		73.58±9.9	
> 24 kg/m^2^	54 (26.7)	198.4±22.2		73.13±12.0	
TNM stage			<0.001		0.012
0–I	77 (38.1)	211.3±14.3		71.12±11.5	
II	109 (54.0)	205.3±19.8		74.61±10.2	
III–IV	16 (7.9)	191.7±20.1		78.56±5.1	
Radiotherapy			<0.001		<0.001
Yes	51 (25.2)	196.0±19.1		78.5±7.2	
No	151 (74.8)	210.1±17.0		71.9±11.1	
Chemotherapy			<0.001		0.416
Yes	125 (61.9)	202.9±19.8		74.1±10.3	
No	77 (38.1)	212.3±14.7		72.8±11.1	
Endocrine therapy			0.579		0.425
Yes	135 (66.8)	207.0±16.8		74.0±10.0	
No	67 (33.2)	205.5±21.7		72.8±11.7	
Neoadjuvant chemotherapy			0.001		<0.001
Yes	47 (23.3)	198.4±20.4		78.5±9.3	
No	155 (76.7)	209.0±17.3		72.1±10.6	
Surgical approach			0.027		0.034
Anterior pectoralis approach	10 (5.0)	193.9±15.8		80.5±6.4	
Posterior pectoralis approach	192 (95.0)	207.2±18.5		73.2±10.7	
Surgical mode			0.133		<0.001
NSM/ssm+ sentinel lymph node dissection + prosthesis implantation	128 (63.4)	208.5±16.8		72.5±10.4	
NSM/ssm+ axillary lymph node dissection + prosthesis implantation	35 (17.3)	202.6±25.2		78.4±9.5	
NSM/ssm+ sentinel lymph node dissection + prosthesis implantation + contralateral breast augmentation	22 (10.9)	207.7±15.9		67.2±11.0	
Latissimus dorsi flap	5 (2.5)	204.0±22.6		78.8±7.4	
Transverse rectus abdominis myocutaneous flap	12 (5.9)	195.9±13.0		81.1±6.3	
Breast volume			0.166		0.940
≤ 200 mL	82 (40.6)	208.8±16.6		73.3±9.6	
201–300 mL	102 (50.5)	205.8±20.2		73.8±11.6	
> 300 mL	18 (8.9)	200.1±16.1		73.7±9.3	
Length of scar			0.116		0.019
>20 cm	185 (91.6)	207.3±18.6		73.0±10.7	
10–20 cm	5 (2.5)	204.0±22.6		78.8±7.4	
<10 cm	12 (5.9)	195.9±13.0		81.1±6.3	
Time of breast reconstruction			0.814		0.549
≤ 1 year	48 (23.8)	207.1±15.9		72.8±10.9	
2–3 years	154 (76.2)	206.3±19.3		73.8±10.5	
Timing of reconstruction			0.277		0.261
Immediate reconstruction	198 (98.0)	206.7±18.6		73.5±10.6	
2-phase reconstruction	4 (2.0)	196.5±10.3		79.5±6.6	
Flap harvesting			0.068		0.014
Yes	16 (7.9)	198.6±16.5		79.9±6.3	
No	185 (91.6)	207.4±18.5		73.1±10.7	
Use of SIS matrix			0.128		0.004
Yes	4 (2.0)	220.5±8.2		88.8±5.9	
No	198 (98.0)	206.2±18.6		73.3±10.5	

NSM: nipple-preserving mastectomy; SSM: skin-preserving mastectomy; HRQOL, health-related quality of life; SIS, small intestinal submucosa.

### Multivariable analysis of satisfaction degree and HRQOL

Age of > 45 years (vs. ≤35 years, *β *=  −  3.74, *P *< 0.001) was an independent risk factor influencing the satisfaction degree score (Table 2). Age between 36 and 45 years (vs. ≤35 years, *β *= − 0.26, *P *< 0.001), age of  > 45 years (vs. ≤35 years, *β *= − 0.45, *P *< 0.001), NSM/SSM + sentinel lymph node dissection + prosthesis implantation + contralateral breast augmentation (vs. NSM/SSM + sentinel lymph node dissection + prosthesis implantation, *β*  = − 0.16, *P *= 0.012), and the use of an SIS matrix (*β *= 0.13, *P *= 0.044) were independent risk factors influencing the HRQOL score (Table 3).

**Table 2:** Multivariable analysis of the satisfaction degree

Variable	Regression coefficient (*B*)	Standardized regression coefficient (*β*)	P
Age			
≤ 35 years	Ref		
36–45 years	− 4.694	− 0.125	0.086
> 45 years	− 15.746	− 3.74	<0.001
Body mass index			
< 18.5 kg/m^2^	Ref		
18.5–24 kg/m^2^	− 3.129	− 0.077	0.676
> 24 kg/m^2^	− 8.633	− 0.206	0.266
TNM stage			
0–I	Ref		
II	− 1.273	− 0.034	0.644
III–IV	− 9.261	− 0.135	0.097
Radiotherapy	− 8.589	− 0.202	0.062
Chemotherapy	− 4.759	− 0.125	0.084
Neoadjuvant chemotherapy	2.742	0.063	0.538
Surgical approach			
Anterior pectoralis approach	Ref		
Posterior pectoralis approach	1.999	0.023	0.722

**Table 3:** Multivariable analysis of the health-related quality of life

Variable	Regression coefficient (*B*)	Standardized regression coefficient (*β*)	*P*
Age			
≤ 35 years	Ref		
36–45 years	− 5.611	− 0.262	<0.001
>45 years	− 10.796	− 0.451	<0.001
TNM stage			
0–I	Ref		
II	6.75	0.032	0.657
III–IV	− 2.300	− 0.059	0.487
Chemotherapy	4.544	1.87	0.090
Neoadjuvant chemotherapy	0.548	0.022	0.840
Surgical approach			
Anterior pectoralis approach	Ref		
Posterior pectoralis approach	1.666	0.034	0.740
Surgical mode			
NSM/SSM+ sentinel lymph node dissection + prosthesis implantation	Ref		
NSM/SSM+ axillary lymph node dissection + prosthesis implantation	1.590	0.057	0.480
NSM/SSM+ sentinel lymph node dissection + prosthesis implantation + contralateral breast augmentation	− 5.441	− 0.161	0.012
Latissimus dorsi flap	13.540	0.200	0.228
Transverse rectus abdominis myocutaneous flap	15.390	0.346	0.118
Flap harvesting	− 7.851	− 0.201	0.461
Use of SIS matrix	9.635	0.128	0.044

NSM: nipple-preserving mastectomy; SSM: skin-preserving mastectomy; SIS, small intestinal submucosa.

## Discussion

This study showed that age of > 45 years was associated with lower satisfaction degree score, while age of ≥36 years, NSM/SSM + sentinel lymph node dissection + prosthesis implantation + contralateral breast augmentation, and the use of SIS matrix were independent factors influencing the HRQOL scores. These results indicated which patients were more likely to achieve a better HRQOL after breast reconstruction and could help improve the management of women with breast cancer.

In this study, age was the only factor related to both satisfaction and HRQOL, with older women being less satisfied with their reconstruction. Complications become more frequent with age, and complications were negatively associated with the mental health score [20]. Still, age should not be a contraindication to breast reconstruction [20]. Girotto et al. [21] also reported that breast reconstruction in older women could help maintain HRQOL but HRQOL could be affected in older women by various physical limitations and comorbidities, while the older women scored better than younger ones regarding the mental outcomes. Ritter et al. [22] also found out that age had a significant impact on QOL after reconstruction but was not a contraindication. Indeed, younger patients have worse QOL outcomes in the social domain because they are often more concerned with their physical appearance and femininity. On the other hand, older patients often see their breast appearance as a less important aspect of their QOL, but they tend to score lower in the physical well-being domains [23].

In this study, the procedure of NSM/SSM, sentinel lymph node dissection, prosthesis implantation, and contralateral breast augmentation was negatively associated with HRQOL. This particular type of surgery involves several procedures that could together increase the morbidity of the intervention and decrease HRQOL. A study showed a different conclusion that lumpectomy or mastectomy before reconstruction did not affect the HRQOL outcomes [24], but it did not consider all procedures regarding the lymph nodes and reconstruction. Additional studies are necessary to further explore the association between various surgery type and HRQOL.

In this study, the use of SIS matrix was positively associated with HRQOL. The use of matrix aims to facilitate one-stage breast reconstruction and create a more natural-looking breast [25]. However, the previous study suggested that using acellular dermal matrix did not appear to affect the HRQOL after reconstruction [26]. Specifically, several matrixes are currently available, and it remains unknown which one could be associated with better outcomes.

A study in Japanese indicated some different factors from our study, such as higher BMI leading to lower “Satisfaction with breasts,” and a bilateral procedure being a significant risk factor for lower “Psychosocial well-being.” Another study from Dartmouth showed complication and surgeon experience were the only independent predictors of lesser improvement of the Satisfaction. A retrospective study showed that factors associated with lower satisfaction included history of psychiatric diagnosis, preoperative radiotherapy, marital status (married), and higher BMI. The discrepancy among the above-related studies suggested the necessity of deeper exploration on the factors associated with patients’ satisfaction.

According to the supplementary tables, “Satisfaction with Breasts,” “Psychosocial Well-being,” “Satisfaction with papilla,” and “Sexual Well-being” showed significant decrease after surgery compared to that before surgery in the overall population as well as in all age subgroups, so breast reconstruction might not bring additional benefits. These findings collectively underscore the multifaceted nature of patient experiences and satisfaction in the context of breast surgery. The observed decreases in certain areas of well-being, such as "Satisfaction with Breasts" and "Psychosocial Well-being," might be attributed to post-surgery adjustments and psychosocial challenges. On the other hand, the increase in "Physical Well-being: Chest" suggests a positive impact on physical comfort following the surgical intervention. The variations in patient satisfaction across different age groups highlight the importance of considering age-related factors when assessing and addressing post-surgery well-being and satisfaction.

This study had several limitations. First, the sample size was not calculated and convenience sampling was used. In addition, the BREAST-Q can delve into intimate matters or raise some emotions. Specifically, when reporting sex-related questions, Chinese women may be very shy and thus hesitate or avoid such questions. Therefore, the sex-related answers in the BREAST-Q might be more or less accurate. Patients feeling distressed when answering the questions could also provide inaccurate answer. Furthermore, the numbers of patients treated using specific techniques for breast surgery, lymph node sampling, and reconstruction were relatively small, and thus the power of the corresponding subgroup analyses was low. Nevertheless, such subgroup analyses can provide directions for future studies.

## Conclusion

In conclusion, older age was associated with lower satisfaction degree in patients receiving breast reconstruction. Older age and the procedure of NSM/SSM, sentinel lymph node dissection, prosthesis implantation, and contralateral breast augmentation were negatively associated with HRQOL, while the use of matrix was positively associated with HRQOL after breast reconstruction in patients with breast cancer. More prospective studies are needed to explore the issues leading to the dissatisfaction of the patients after breast reconstruction and thus to improve the surgical mode and details.

### Supplementary Information

Below is the link to the electronic supplementary material.

Supplementary file1 (DOC 52 kb)

## References

[1] Sung H, Ferlay J, Siegel RL, Laversanne M, Soerjomataram I, Jemal A (2021). Global cancer statistics 2020: GLOBOCAN estimates of incidence and mortality worldwide for 36 cancers in 185 countries. CA Cancer J Clin.

[2] Siegel RL, Miller KD, Fuchs HE, Jemal A (2022). Cancer statistics, 2022. CA Cancer J Clin.

[3] National Comprehensive Cancer Network (2022) NCCN Clinical Practice Guidelines in Oncology (NCCN Guidelines). Breast Cancer. Version 4.2022. Fort Washington

[4] Munhoz AM, Montag E, Filassi JR, Gemperli R (2014). Current approaches to managing partial breast defects: the role of conservative breast surgery reconstruction. Anticancer Res.

[5] Ettridge K, Scharling-Gamba K, Miller C, Roder D, Prichard I (2022). Body image and quality of life in women with breast cancer: appreciating the body and its functionality. Body Image.

[6] Brunet J, Price J, Harris C (2022). Body image in women diagnosed with breast cancer: a grounded theory study. Body Image.

[7] Platt J, Baxter N, Zhong T (2011). Breast reconstruction after mastectomy for breast cancer. CMAJ.

[8] Fann JR, Thomas-Rich AM, Katon WJ, Cowley D, Pepping M, McGregor BA (2008). Major depression after breast cancer: a review of epidemiology and treatment. Gen Hosp Psychiatry.

[9] Hegel MT, Moore CP, Collins ED, Kearing S, Gillock KL, Riggs RL (2006). Distress, psychiatric syndromes, and impairment of function in women with newly diagnosed breast cancer. Cancer.

[10] Ahn SH, Park BW, Noh DY, Nam SJ, Lee ES, Lee MK (2007). Health-related quality of life in disease-free survivors of breast cancer with the general population. Ann Oncol.

[11] Trejo-Ochoa JL, Maffuz-Aziz A, Said-Lemus FM, Dominguez-Reyes CA, Hernández-Hernández B, Villegas-Carlos F (2013). Impact on quality of life with breast reconstructive surgery after mastectomy for breast cancer. Ginecol Obstet Mex.

[12] Marroquín B, Czamanski-Cohen J, Weihs KL, Stanton AL (2016). Implicit loneliness, emotion regulation, and depressive symptoms in breast cancer survivors. J Behav Med.

[13] Fanakidou I, Zyga S, Alikari V, Tsironi M, Stathoulis J, Theofilou P (2018). Mental health, loneliness, and illness perception outcomes in quality of life among young breast cancer patients after mastectomy: the role of breast reconstruction. Qual Life Res.

[14] Saldanha IJ, Cao W, Broyles JM, Adam GP, Bhuma MR, Mehta S et al (2021) Breast reconstruction after mastectomy: a systematic review and meta-analysis. AHRQ Comparative Effectiveness Reviews, Rockville, MD 34383395

[15] Pusic AL, Klassen AF, Scott AM, Klok JA, Cordeiro PG, Cano SJ (2009). Development of a new patient-reported outcome measure for breast surgery: the BREAST-Q. Plast Reconstr Surg.

[16] Seth I, Seth N, Bulloch G, Rozen WM, Hunter-Smith DJ (2021). Systematic review of breast-Q: A tool to evaluate post-mastectomy breast reconstruction. Breast Cancer (Dove Med Press).

[17] Liu LQ, Branford OA, Mehigan S (2018). BREAST-Q measurement of the patient perspective in oncoplastic breast surgery: a systematic review. Plast Reconstr Surg Glob Open.

[18] Li JB, Jiang ZF (2021). Chinese society of clinical oncology breast cancer guideline version 2021: updates and interpretations. Zhonghua Yi Xue Za Zhi.

[19] Ma Y, Xiu B, Shao D (2021). Development of a Chinese version of BREAST-Q and a retrospective study of its evaluation of patients’ satisfaction in breasts. Zhong Guo Shi Yong Wai Ke Za Zhi

[20] Bowman CC, Lennox PA, Clugston PA, Courtemanche DJ (2006). Breast reconstruction in older women: should age be an exclusion criterion?. Plast Reconstr Surg.

[21] Girotto JA, Schreiber J, Nahabedian MY (2003). Breast reconstruction in the elderly: preserving excellent quality of life. Ann Plast Surg.

[22] Ritter M, Ling BM, Oberhauser I, Montagna G, Zehnpfennig L, Lévy J (2021). The impact of age on patient-reported outcomes after oncoplastic versus conventional breast cancer surgery. Breast Cancer Res Treat.

[23] Cimprich B, Ronis DL, Martinez-Ramos G (2002). Age at diagnosis and quality of life in breast cancer survivors. Cancer Pract.

[24] Freitas-Silva R, Conde DM, de Freitas-Júnior R, Martinez EZ (2010). Comparison of quality of life, satisfaction with surgery and shoulder-arm morbidity in breast cancer survivors submitted to breast-conserving therapy or mastectomy followed by immediate breast reconstruction. Clinics (Sao Paulo).

[25] Salzberg CA (2006). Nonexpansive immediate breast reconstruction using human acellular tissue matrix graft (AlloDerm). Ann Plast Surg.

[26] Lohmander F, Lagergren J, Johansson H, Roy PG, Frisell J, Brandberg Y (2020). Quality of life and patient satisfaction after implant-based breast reconstruction with or without acellular dermal matrix: randomized clinical trial. BJS Open.

