# Supplementary material for: BREAST-Q-Based Survey of the Satisfaction and Health Status of Patients with Breast Reconstruction
Source: Aesthetic Plast Surg. 2023 Sep 11;47(6):2295–303. doi: 10.1007/s00266-023-03642-2 (PMC10784367; doi:10.1007/s00266-023-03642-2)
Supplement: Supplementary file 1 — Supplementary file1 (DOC 52 kb) [file 266_2023_3642_MOESM1_ESM.doc]

**Supplementary Table 1.** The comparison of satisfaction degree score between before and after surgery

|  | Before surgery | After surgery | P |
| --- | --- | --- | --- |
| Satisfaction with Breasts | 59.06±1.31 | 45.95±9.70 | <0.001 |
| Psychosocial Well-being | 48.19±3.16 | 39.59±6.03 | <0.001 |
| Physical Well-being: Chest | 11.32±0.64 | 12.93±1.62 | <0.001 |
| Satisfaction with papilla | 4.00±0 | 3.24±0.84 | <0.001 |
| Sexual Well-being | 26.84±4.26 | 18.5±5.95 | <0.001 |
| Satisfaction with the prosthesis | - | 7.61±0.84 | - |
| Satisfaction with the information | - | 50.09±5.62 | - |
| Satisfaction with the surgeons | - | 45.25±3.26 | - |
| Satisfaction with the medical team | - | 26.72±1.68 | - |
| Satisfaction with the other medical staff | - | 27.22±1.34 | - |

**Supplementary Table 2.** The comparison of satisfaction degree score between before and after surgery among the age subgroups

|  | ≤35 years old | | | 35-45 years old | | | >45 years old | | | P^#^ | P^&^ |
| --- | --- | --- | --- | --- | --- | --- | --- | --- | --- | --- | --- |
|  | Before surgery | After surgery | P^*^ | Before surgery | After surgery | P^*^ | Before surgery | After surgery | P^*^ |
| Satisfaction with Breasts | 59.71±0.95 | 50.23±7.23 | <0.001 | 59.20±1.21 | 45.64±9.66 | <0.001 | 58.06±1.28 | 41.17±10.22 | <0.001 | <0.001 | <0.001 |
| Psychosocial Well-being | 49.68±1.48 | 42.14±6.4 | <0.001 | 49.06±2.11 | 39.55±5.55 | <0.001 | 44.98±3.79 | 36.53±4.82 | <0.001 | <0.001 | <0.001 |
| Physical Well-being: Chest | 11.02±0.12 | 12.42±0.93 | <0.001 | 11.18±0.44 | 12.92±1.8 | <0.001 | 11.92±0.85 | 13.58±1.77 | <0.001 | <0.001 | <0.001 |
| Satisfaction with papilla | 4±0 | 3.43±0.77 | <0.001 | 4±0 | 3.21±0.82 | <0.001 | 4±0 | 3.04±0.94 | <0.001 | - | 0.040 |
| Sexual Well-being | 29.94±0.5 | 21.98±6.24 | <0.001 | 27.26±3.98 | 18.26±5.26 | <0.001 | 22.38±3.44 | 14.6±3.76 | <0.001 | <0.001 | <0.001 |
| Satisfaction with the prosthesis | - | 7.78±0.72 | - | - | 7.58±0.87 | - | - | 7.43±0.91 | - | - | 0.073 |
| Satisfaction with the information | - | 51.32±5.22 | - | - | 50.39±5.78 | - | - | 48.09±5.4 | - | - | 0.006 |
| Satisfaction with the surgeons | - | 46.4±2.51 | - | - | 45.61±3.2 | - | - | 43.28±3.35 | - | - | <0.001 |
| Satisfaction with the medical team | - | 27.25±1 | - | - | 26.93±1.6 | - | - | 25.75±2.06 | - | - | <0.001 |
| Satisfaction with the other medical staff | - | 27.6±0.92 | - | - | 27.26±1.51 | - | - | 26.7±1.34 | - | - | 0.001 |

^*^ paired-samples t test

^#^ One-way ANOVA test, compare the data before surgery data among three age subgroups.

^&^ One-way ANOVA, compare the after surgery data among three age subgroups.
